# Real‐Time Bladder Perfusion Assessment Using ICG Fluorescence Imaging Enables Successful Reconstruction

**DOI:** 10.1002/iju5.70116

**Published:** 2025-11-09

**Authors:** Kosuke Iwatani, Mahito Atsuta, Yuma Okamoto, Kazuhiro Takahashi, Yu Imai, Fumihiko Urabe, Takahiro Kimura, Jun Miki

**Affiliations:** ^1^ Department of Urology The Jikei University School of Medicine, Kashiwa Hospital Chiba Japan; ^2^ Department of Urology The Jikei University School of Medicine Tokyo Japan

**Keywords:** bladder ischemia, bladder‐sparing, indocyanine green

## Abstract

**Introduction:**

We report a case where intraoperative indocyanine green (ICG) imaging was utilized to evaluate bladder perfusion, enabling successful bladder preservation and bilateral ureteral reimplantation.

**Case Presentation:**

A 49‐year‐old woman underwent resection of a recurrent pelvic tumor. The tumor involved both ureters and bilateral vesical arteries, which were sacrificed during surgery. ICG fluorescence imaging demonstrated uniform bladder wall perfusion, guiding the decision to proceed with bladder preservation. Postoperatively, contrast‐enhanced CT and cystoscopy confirmed preserved bladder perfusion and mucosal integrity. The patient voided spontaneously without dysfunction.

**Conclusion:**

Real‐time evaluation of bladder perfusion using ICG fluorescence enabled safe bladder preservation in a situation where major vascular supply was compromised. This technique may assist in intraoperative decision‐making during complex pelvic surgeries.

**Abbreviation**
ICG: indocyanine Green

**Summary**

- This case highlights the clinical utility of real‐time perfusion assessment using indocyanine green (ICG) fluorescence imaging during bladder reconstruction.
- The technique allowed surgeons to identify poorly perfused areas intraoperatively and to modify the reconstructive plan accordingly.
- This approach may reduce the risk of postoperative complications and facilitate bladder preservation in selected patients.

## 1 Introduction

Indocyanine green (ICG) is a fluorescent dye that enables real‐time visualization of tissue perfusion under near‐infrared imaging. ICG has since gained widespread use in surgical disciplines, particularly for vascular and perfusion assessment [1, 2]. In urologic surgery, ICG has been used in various settings, such as partial nephrectomy, identification of sentinel lymph nodes of the prostate, and evaluation of ureteral vascularity during reconstructive procedures [3, 4, 5, 6]. Despite its growing use in the upper urinary tract, the application of ICG for bladder perfusion assessment remains extremely limited. To our knowledge, there have been no previous reports describing intraoperative evaluation of bladder blood perfusion using ICG fluorescence.

Here, we present a case in which ICG fluorescence imaging was utilized to assess bladder perfusion after resection of bilateral vesical arteries, enabling successful bladder preservation and bilateral ureteral reimplantation.

## 2 Case Presentation

A 49‐year‐old female with a history of radical hysterectomy and pelvic lymphadenectomy for cervical cancer underwent open resection of a recurrent pelvic tumor. Intraoperatively, the tumor showed strong adhesion to the vaginal stump, rectum, bilateral lower ureters, and internal iliac arteries. Therefore, low anterior resection and bilateral distal ureteral resection were performed. During tumor removal, the right internal iliac artery and the left superior and inferior vesical arteries were transected.

**FIGURE 1:**
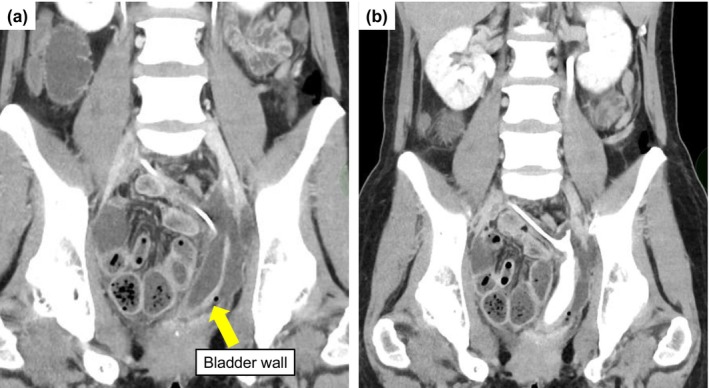
Postoperative contrast‐enhanced CT of the bladder. (a) Early phase demonstrated enhancement of the entire bladder wall. (b) The bladder was deviated to the left side without no obvious urinary leakage was observed.

Major blood supply to the bladder was sacrificed; there was a high risk of postoperative bladder ischemia and necrosis. After confirming sufficient perfusion of the bladder and ureters using ICG fluorescence imaging, bilateral ureteral reimplantation was performed. Postoperatively, a urinary catheter and both ureteral stents were removed 1 month later. Follow‐up contrast‐enhanced CT demonstrated enhancement of the entire bladder wall, and cystoscopic findings revealed well‐preserved mucosal color and integrity (Figure 1). Although the patient experienced diminished bladder sensation following bilateral hypogastric nerve resection, voided volume per micturition was preserved, without evidence of significant post‐void residual, hydronephrosis, or renal dysfunction.

### 2.1 Intraoperative Evaluation of Bladder Perfusion Using ICG

**FIGURE 2:**
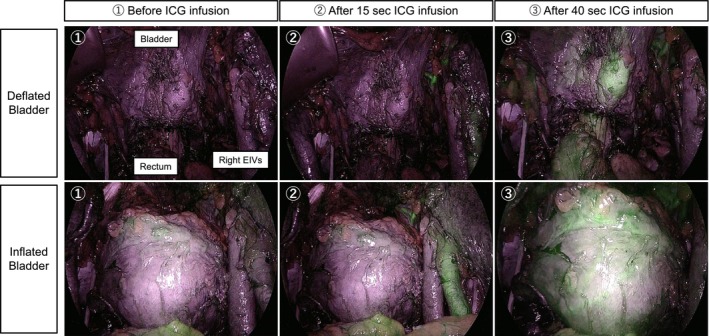
Bladder vascular perfusion using ICG fluorescence imaging. Bladder perfusion visualized with near‐infrared fluorescence imaging at (1) before ICG injection, (2) 15 s after ICG injection, and (3) 40 s after ICG injection. During bladder deflation, perfusion is less visible due to compression by the peritoneum and surrounding tissues. In contrast, bladder inflation allows clear visualization of the entire bladder wall perfusion.

Since the main blood vessels supplying the bladder were severed bilaterally, cystectomy was initially considered as a safer option. However, bladder preservation was attempted due to the patient's young age and the absence of a need for bowel diversion. Intraoperative near‐infrared fluorescence imaging was performed using intravenous ICG (Diagnogreen contains 25 mg of ICG per ampule, Daiichi Sankyo Co. Ltd., Tokyo, Japan) and the VISERA ELITE II system (Olympus Corporation, Tokyo, Japan). Intraoperative assessment of bladder perfusion using ICG fluorescence imaging was first performed with the bladder in a deflated state, but adequate evaluation was difficult due to interference from surrounding fat, peritoneum, and bleeding. When the bladder was inflated with 250 mL of saline, repeat ICG imaging showed uniform staining of the entire bladder wall without laterality approximately 30 s after injection, indicating that sufficient perfusion was maintained (Figure 2).

### 2.2 Surgical Procedure

**FIGURE 3:**
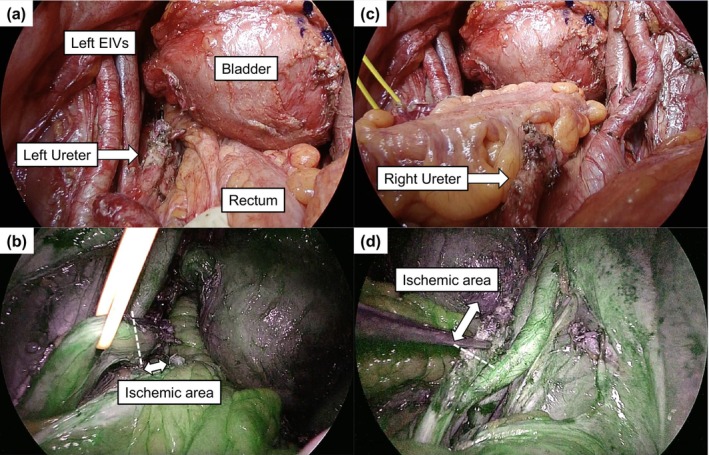
Bilateral ureters vascular perfusion using ICG fluorescence imaging. Intraoperative ICG fluorescence evaluation of bilateral ureters. At 40–50 s after ICG injection, poor perfusion was observed at the distal ends, and these ischemic area segments were resected.

**FIGURE 4:**
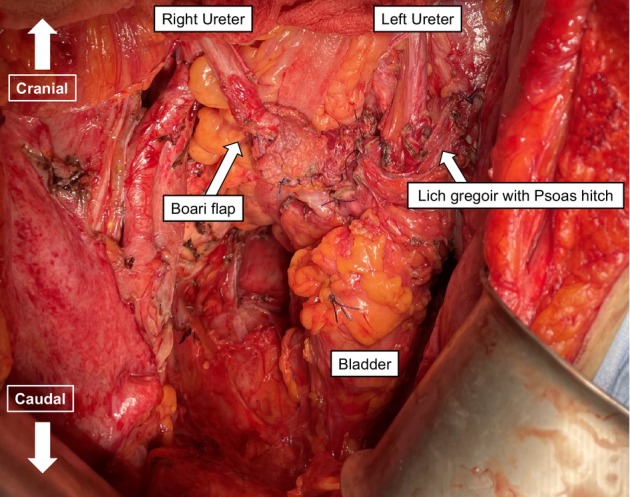
Bilateral ureteroneocystostomy. The Lich‐Gregoir technique with a psoas hitch (left) and vesicoureteral reimplantation using a Boari flap (right) were performed to ensure that each ureter remains tension‐free, even when the bladder is deflated.

The both ureters were transected at their crossing with the common iliac artery. As bilateral psoas hitch procedures were not feasible, ureteral reimplantation was performed in a stepwise manner. The ureteral ends were resected up to segments where sufficient blood flow was confirmed using ICG fluorescence imaging (Figure 3). The longer left ureter was first anastomosed using the Lich‐Gregoir technique with a psoas hitch, followed by right ureteral reimplantation using a Boari flap (Figure 4). A 4‐cm square bladder flap was created to allow tension‐free anastomosis of both ureters even after bladder deflation and creation of a submucosal tunnel. Both anastomoses were performed with 4‐0 absorbable sutures.

## 3 Discussion

This is the first report to assess bladder perfusion intraoperatively using ICG fluorescence imaging. In this case, real‐time evaluation of bladder blood flow allowed us to avoid cystectomy.

The bladder is primarily supplied from the internal iliac artery. The bladder is mainly supplied by vesical arteries and collateral pelvic branches. When both internal iliac arteries are acutely disrupted, such as after pelvic embolization for trauma, rapid loss of bladder perfusion may lead to bladder necrosis or anastomotic failure [7]. These complications may require long‐term catheter management or urinary diversion [8]. Therefore, intraoperative real‐time assessment of residual blood flow to pelvic organs, including the bladder, is essential to guide decision‐making and prevent such adverse outcomes.

In urologic surgery, the utility of ICG imaging for ureteroneocystostomy has been reported [5]. Hebert et al. reported that ICG imaging of ureteral perfusion led to additional resection of ischemic ureteral segments in 63% of cases, and the rate of ureteral stricture was as low as 1.8%, suggesting the importance of preserving adequate vascular supply for optimal urinary tract function [4].

Because ICG remains intravascular, it is suitable for evaluating superficial blood flow but has limitations in assessing deeper tissue and mucosal perfusion. In addition, perivesical structures may scatter or obscure the fluorescence signal. To address these limitations, we performed perfusion assessment with the bladder inflated. Since blood flow in the bladder is generally from the outer to the inner layers and intramural vessels communicate extensively, this approach may reasonably reflect the overall perfusion status.

There are no published data regarding appropriate dose, the intensity or time to fluorescence in the bladder following ICG injection. However, since the bladder receives direct branches from the internal iliac artery, it is assumed to be similar to other pelvic organs. In rectal surgery, doses of 0.2–0.5 mg/kg are commonly used, but in this case, multiple assessments were required [1]. Therefore, we used 0.1 mg/kg per injection and confirmed complete washout before each subsequent evaluation. Regarding fluorescence intensity and time to fluorescence, delayed fluorescence beyond 31 s has been associated with a higher risk of anastomotic leakage in rectal surgery [9]. In our case, bladder wall fluorescence was most clearly visible around 40 s after injection. This was comparable to the fluorescence intensity observed in the adjacent colon, and we considered that the lower ICG dose may have contributed to this delay. Since the bladder is mainly composed of muscle tissue, uniform staining of the entire wall may be important for maintaining normal voiding function. Further investigation is needed to determine whether fluorescence intensity and perfusion time affect healing and function in the bladder.

Considering that both vesical arteries had been sacrificed, more cautious assessment, such as additional evaluations immediately after creating the psoas hitch flap or completing the ureteral anastomoses, might have been warranted. In future cases, additional ICG imaging at these critical timepoints may help ensure flap viability and anastomotic safety.

## 4 Conclusion

Intraoperative ICG fluorescence imaging was successfully used to assess bladder perfusion following bilateral vesical artery resection. This technique allowed for safe bladder preservation and ureteral reimplantation, and may be a useful tool for real‐time evaluation of urinary tract perfusion in complex pelvic surgeries.

## Consent

Written informed consent was obtained from the patient for publication of this case report and accompanying images.

## Conflicts of Interest

The authors declare no conflicts of interest.

## Data Availability

The data that support the findings of this study are available on requssest from the corresponding author. The data are not publicly available due to privacy or ethical restrictions.
